# Characterization of Porcine Ventral Mesencephalic Precursor Cells following Long-Term Propagation in 3D Culture

**DOI:** 10.1155/2012/761843

**Published:** 2012-11-20

**Authors:** Pia S. Jensen, Lise Lyck, Pia Jensen, Jens Zimmer, Morten Meyer

**Affiliations:** ^1^Department of Neurobiology Research, Institute of Molecular Medicine, University of Southern Denmark, 5000 Odense C, Denmark; ^2^Coloplast Danmark, Holtedam 1-3, 3050 Humlebæk, Denmark

## Abstract

The potential use of predifferentiated neural precursor cells for treatment of a neurological disorder like Parkinson's disease combines stem cell research with previous experimental and clinical transplantation of developing dopaminergic neurons. One current obstacle is, however, the lack of ability to generate dopaminergic neurons after long-term *in vitro* propagation of the cells. The domestic pig is considered a useful nonprimate large animal model in neuroscience, because of a better resemblance of the larger gyrencephalic pig brain to the human brain than the commonly used brains of smaller rodents. In the present study, porcine embryonic (28–30 days), ventral mesencephalic precursor cells were isolated and propagated as free-floating neural tissue spheres in medium containing epidermal growth factor and fibroblast growth factor 2. For passaging, the tissue spheres were cut into quarters, avoiding mechanical or enzymatic dissociation in order to minimize cellular trauma and preserve intercellular contacts. Spheres were propagated for up to 237 days with analysis of cellular content and differentiation at various time points. Our study provides the first demonstration that porcine ventral mesencephalic precursor cells can be long-term propagated as neural tissue spheres, thereby providing an experimental 3D *in vitro* model for studies of neural precursor cells, their niche, and differentiation capacity.

## 1. Introduction

Parkinson's disease (PD) is characterized by a progressive loss of dopaminergic neurons in the substantia nigra in the ventral mesencephalon (VM). The disease is clinically characterized by serious motor dysfunctions, including muscle rigidity, tremor, bradykinesia, and sometimes akinesia [1]. A number of experimental animal and clinical studies using human fetal VM tissue have suggested that intrastriatal transplantation of dopaminergic neurons may be an effective treatment for patients with PD [2–4]. However, low availability of human foetal tissue, ethical concerns related to its procurement and use, limited survival and integration of the grafted dopaminergic neurons, and the highly variable developmental stage of the donor tissue have restricted the clinical application [5].

A number of studies have characterized two neurogenic areas in the mammalian brain, the subventricular zone (SVZ) lining the lateral ventricles [6] and the dentate gyrus of the hippocampus [7]. The existence of precursor cells with neurogenic potential has also been reported for the developing [8–10] and to some degree the adult [11–15] ventral midbrain. Such neural precursor cells expanded *in vitro* are considered a potential source of cells for cell replacement in PD [16]. However, generation of functional dopaminergic neurons from this cell source has proven difficult [17], and therefore there is a need to develop experimental protocols and models for studies of precursor cell proliferation and dopaminergic differentiation.

Usually cultures of neural precursor cells are established by initial mechanical or enzymatic dissociation as well as enzymatic dissociation during cell passaging. The enzymatic dissociation may lead to stripping of proteins on the cell surface and in this way disrupt pathways for intracellular signal transduction, intercellular communication, and cell migration, which are important for cell development, proliferation, and differentiation [18]. In order to avoid mechanical and enzymatic dissociation and to preserve an organotypic intercellular microenvironment during propagation of neural precursor cells, culturing of tissue microexplants as neural tissue spheres (NTS) was established in our laboratory [19, 20]. In contrast to the classical cell-aggregate neurospheres [21, 22], cells in NTS cultures are never dissociated, neither at the time of tissue sampling and culture set up nor at cell passaging.

The domestic pig (*Sus scrofa domesticus*) is considered a useful nonprimate model in neuroscience because of a better resemblance of the gyrencephalic pig brain to the human brain in the general anatomy, growth, and development compared to the lissencephalic brains of small mammals such as rodents [23–25].

In the present paper, we report for the first time data on efficient long-term propagation of NTS derived from fetal porcine VM.

## 2. Experimental Procedures

### 2.1. Isolation of Ventral Mesencephalic Tissue

Pig embryos (Danish Landrace × Yorkshire × Duroc; Biomedical Laboratory, University of Southern Denmark, DK) of gestational age E28–30 (crown to rump length = 25–27 mm) were used. The embryos were removed by hysterectomy from sows (5–9 embryos/preparation; two independent experiments), terminally anesthetized with an intravenous overdose of pentobarbital, and placed in sterile 0.9% salt solution until removal of the brains. The animal procedures were approved by and conducted in compliance with The Danish Animal Health Care Committee (J. no. 192000/561-272).

**Figure 1:**
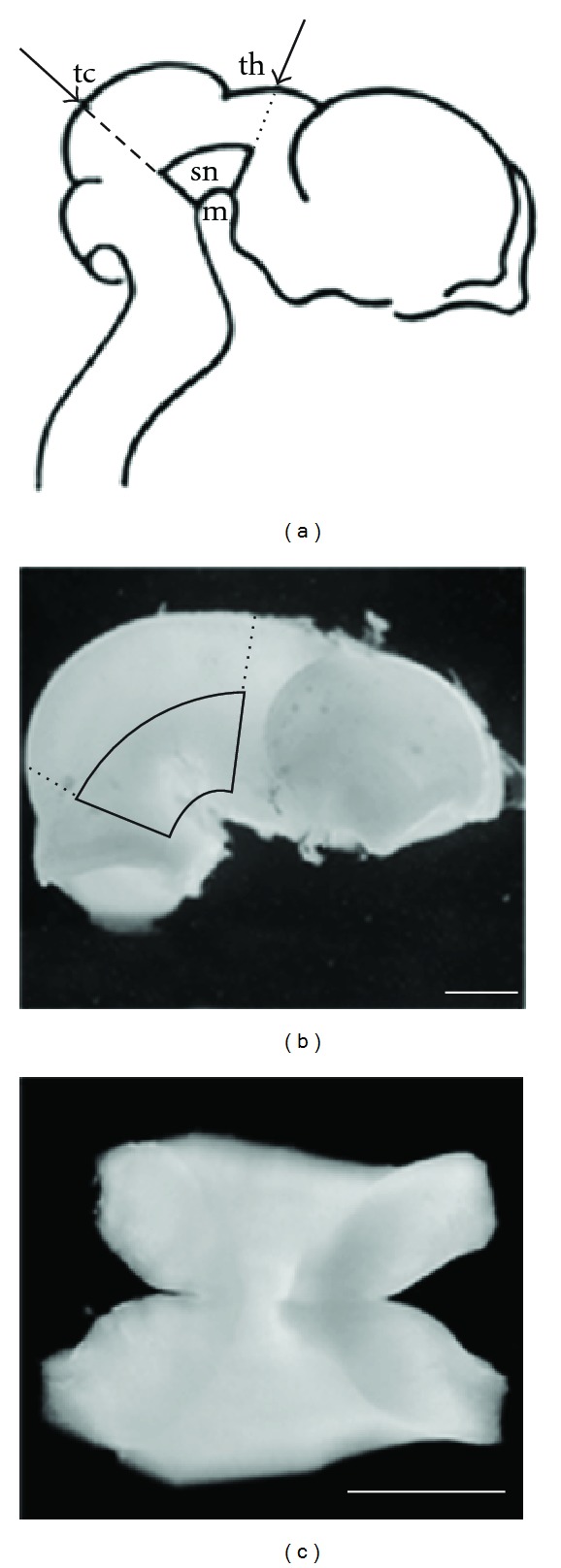
Dissection of porcine embryonic (E28–30) ventral mesencephalon (VM). (a) Lateral perspective showing the angle of the first two cuts of the dissection, with imaginary lines (arrows) intersecting the dorsal surface of the tectum (tc) and the thalamus (th) (from Dunnett and Bjorklund, 1992). (b) Brain of a 28-day-old embryo with the dissection lines from (a) marked. The area encircled by solid lines represents the VM. (c) The ventral surface of the isolated VM explant. Scale bars: 500 *μ*m.

Brains were removed from the skull and placed in chilled Gey's balanced salt solution (GIBCO) with 0.5% D-glucose under sterile conditions. Then, the meninges were gently removed, and the ventral mesencephali isolated by rostral, caudal, lateral, and dorsal cuts (Figure 1). 

With the purpose of generating reference material for immunohistochemical staining and characterization of neuronal and glial cell types, VM tissue blocks were fixed in 4% paraformaldehyde (PFA) in 0.15 M phosphate buffer immediately after isolation.

### 2.2. Setup and Propagation of VM Neural Tissue Spheres (NTS)

Following microscopical inspection, the VM explants were further trimmed for excess tissue and cut into small tissue blocks either by manual division of the tissue blocks into quarters using a scalpel knife or by sectioning the tissue blocks at 350 *μ*m using a McIlwain tissue chopper. The resulting tissue blocks were cultured as free-floating NTS in plastic flasks (NUNC) placed in a humidified incubator atmosphere at 36°C and 5% CO_2_.

For culturing, two different serum-free mitogen-containing media were tested. The first named *neurosphere- (NS-) proliferation medium* is normally used for our rat stem cell cultures [19] and was composed of a 1 : 1 mixture of Dulbecco's Modified Eagles Medium (DMEM) and Nut Mix.F12 (Gibco 21331-020) added 0.6% (w/v) glucose (Sigma), 5 mM Hepes (Gibco), 20 mM NaHCO_3_ (Sigma), 1% bovine serum albumin (BSA; Sigma), 1% Non-Essential Amino Acids (NEAA; Sigma), 1% N2 supplement (Gibco), 4 mM L-glutamine (Gibco), 1% Pen/Strep, 20 ng/mL human recombinant Epidermal Growth Factor (EGF; R&D Systems, 236-EG), and 20 ng/mL human recombinant basic Fibroblast Growth Factor (bFGF; R&D Systems, 233-FB). The second medium, Human Neural Stem Cell *(HNSC-) proliferation medium,* is normally used for human neural stem cell cultures [26] and was composed of a 1 : 1 mixture of Dulbeccos Modified Eagles Medium (DMEM) and Nut Mix.F12 with Glutamax (Gibco 31331-028) added 0.6% (w/v) glucose (Sigma), 5 mM Hepes (Gibco), 0.5% (v/v) AlbuMAX-1 (Gibco), 1% Non-Essential Amino Acids (NEAA; Sigma), 1% N2 supplement (Gibco), 1% Pen/Strep (Gibco), 20 ng/mL EGF, and 20 ng/mL bFGF. Two-thirds of the medium was replaced twice a week.

When the NTS had reached a diameter of approximately 600–800 *μ*m, the NTS were passaged (passage 1 = P1) by manually cutting them under microscopical guidance into quarters using a scalpel knife. Based on the size criteria (see above), the manually cut NTS were first passaged every 6–8 days (P0–P3), then every 10–14 days (P3–P10) and 15–22 days (P10–P22), while the NTS cut by a tissue chopper first were passaged every 20–23 days (P0–P3), then every 29 days (P3–P5), and finally every 39 days (P5-P6).

In the subsequent propagation and differentiation experiments, we did not distinguish between NTS manually cut and NTS processed by a McIlwain tissue chopper.

The NS-proliferation medium was found not to support long-term proliferation of porcine VM NTS. Therefore, cultures attempted grown in this medium were early on excluded from the study.

### 2.3. Monitoring of Cell Proliferation in Propagated NTS

For nuclear labelling of proliferating cells in the expanded NTS, 100 *μ*M Bromodeoxyuridine (BrdU; Sigma) was added to the mitogen-containing proliferation medium for 24 h followed by fixation of the cultures in 4% PFA in 0.15 M phosphate buffer.

### 2.4. Induced Differentiation of VM NTS after 51–232 Days of Propagation

The factors acidic fibroblast growth factor (aFGF), forskolin, phorbol 12-myristate 13-acetate (TPA), and N^6^,2′-O-dibutyryladenosine 3′ : 5′-cyclic monophosphate sodium (db-cAMP) stimulate dopaminergic differentiation in neural rat (our laboratory; unpublished) or human [27] forebrain cultures (*in vitro* forebrain tyrosine hydroxylase- (TH-) inducing factors), while fibroblast growth factor 8b (FGF-8b) and sonic hedgehog (SHH) are essential signalling molecules in the embryonic development of nigral dopaminergic neurons (*in vivo* midbrain TH-inducing factors) [28].

To induce dopaminergic differentiation of cells propagated as NTS for 51–232 DIV, NTS were plated onto laminin (20 *μ*g/mL; Sigma) and fibronectin-precoated (20 *μ*g/mL; Invitrogen) 6-well plastic plates and the hitherto used proliferation medium replaced by (1) pure mitogen-free HNSC medium, (2) mitogen-free HNSC medium supplemented by 100 ng/mL human recombinant aFGF (Sigma, F5542), 25 *μ*M forskolin (Sigma, F6886), 100 nM TPA (Sigma, P8139), and 100 *μ*M db-cAMP (Sigma D0627) (TH-inducing factors), or (3) mitogen-free HNSC medium supplemented by 100 ng/mL mouse recombinant FGF-8b (R&D Systems, 423-F8) and 25 ng/mL mouse recombinant SHH (R&D Systems, 461-SH). The series of VM NTS were then grown for 10 or 25 days with complete change of media every 4 days.

As a positive control experiment for validation of the biological activity of aFGF, forskolin, TPA, and db-cAMP, a series of pig forebrain NTS cultures, derived from the SVZ of embryos E28–30 days of age (essentially as described by Andersen et al. [19]), were included in the differentiation step parallel to the VM NTS cultures. These SVZ NTS were propagated and selected by the same criteria and methods as described for the VM NTS.

### 2.5. Histological Processing

Porcine embryonic VM tissue (E28–30) and propagated VM NTS were fixed in 4% PFA in 0.15 M phosphate buffer at room temperature (RT) for 30 min and cryoprotected in 0.15 M phosphate buffer containing 20% sucrose at 4°C. The tissue was embedded in Tissue-Tek (AX-LAB a/s), frozen in CO_2_, and stored at −20°C until cryostat sectioning at 20 *μ*m. Sections were mounted on gelatinised or Superfrost Plus (BrdU labelled NTS) glass slides and stored at −20°C until immunocytochemical staining. Differentiated NTS (*n* = 6–18/group/immunostaining) were fixed in 4% PFA in 0.15 M phosphate buffer for 20 min, rinsed in 0.15 M phosphate buffer, and kept at 4°C in the same buffer until further processing.

### 2.6. Immunocytochemistry

The three-step Biotin-avidin Horse Radish Peroxidase procedure was used. In brief, all VM cryosections, NTS cryosections, and differentiated NTS were adjusted to RT, rinsed for 3 × 15 min in 0.05 M Tris buffered saline (TBS; pH 7.4) containing 1% Triton X-100 (TBS-T), blocked with 10% fetal bovine serum (FBS) in 0.05 M TBS for 30 min at RT, and incubated overnight at 4°C with one of the following primary antibodies in 10% FBS in 0.05 M TBS: anti-Ki67, anti-neuroepithelial stem cell protein (Nestin), anti-Pax6, anti-MASH1, anti-*β* Tubulin III, anti-neuroepithelial associated protein 2 (MAP2), anti-neuronal nuclei (NeuN), anti-tyrosine hydroxylase (TH), anti-Glial Fibrillary Acidic Protein (GFAP), anti-Vimentin, or anti-2′,3′-cyclic nucleotide 3′-phosphodiesterase (CNPase) (see Supplementary Table 1 in Supplementary material available online at doi:10.1155/2012/761843). The specimens were subsequently transferred to RT, rinsed for 3 × 15 min in 0.05 M TBS-T, and incubated for 1 h at RT with the appropriate secondary antibody, biotinylated sheep anti-mouse (Amersham, RPN1001), or biotinylated donkey anti-rabbit (Amersham, RPN1004) diluted 1 : 200 in 10% FBS in 0.05 M TBS. Next, specimens were rinsed for 3 × 15 min in 0.05 M TBS-T and incubated for 1 h at RT with horseradish-peroxidase- (HRP-) conjugated streptavidin (DAKO, P0397) diluted 1 : 200 in 10% FBS in 0.05 M TBS. After further rinsing for 3 × 15 min in 0.05 M TBS, the HRP was visualized by exposure to 0.05% diaminobenzidine (DAB) and 0.033% H_2_O_2_ dissolved in 0.05 M TBS. Samples were then rinsed 3 × 15 min in 0.05 M TBS, and VM and NTS cryosections were dehydrated in ethanol (70, 96, 99%, 2 × 5 min each), cleared in xylene (3 × 5 min), and cover-slipped in DePex (BDH). Differentiated NTS were cover-slipped in Aquatex (VWR) immediately after the final rinse.

For BrdU immunocytochemistry, NTS cryosections were adjusted to RT, rinsed for 3 × 15 min in 2 × Saline Sodium Citrate (SSC; Merck), incubated for 2 h in 2 × SSC/49% formamide (Bie & Berntsen) at 60°C, rinsed in 2 × SSC for 2 × 5 min at 60°C, incubated in 2 N HCL/0.05 M TBS for 30 min at 37°C, and rinsed for 10 min in 0.1 M Borate buffer (Merck, pH 8.5) followed by 15 min in 0.05 M TBS. Thereafter the sections were subjected to the standard three-step Biotin-avidin Horse Radish Peroxidase procedure as described above, using a mouse anti-BrdU antibody (DAKO; M0744) diluted 1 : 200 (Supplementary Table 1).

For control of specificity, the primary antibody was either omitted or substituted in equivalent concentrations with either isotype-specific mouse IgG_1_ (DAKO, X0931), mouse IgG_2a_ (DAKO, X0943), or rabbit IgG fraction (DAKO, X0903). A Leica DC camera fitted to a Zeiss Axiophot microscope was used for acquiring photomicrographs for documentation.

### 2.7. Cell Counting and Statistical Analysis

Quantification of Ki67-positive and BrdU-positive cells in NTS was performed on 20 *μ*m cryostat sections. Images were obtained at 100x magnification. A grid, dividing the NTS into quarters, was overlaid, and Ki67-positive or BrdU-positive cells were counted in one square (representing 25% of the section area) using the cell counting tool of FluoroView 2.1b (Olympus). The area of each NTS section was measured by delineating the NTS using CAST Grid system software (Olympus). Data are presented as average number of cells per mm^2^  ± SEM.

Statistical comparison was performed by means of commercially available software (InStat, GraphPad software). Densities of Ki67-positive or BrdU-positive cells were compared by the parametric one-way analysis of variance (ANOVA) followed by Student-Newman-Keuls multiple comparison test. Differences were considered statistically significant at *P* < 0.05 indicated by *, *P* < 0.01 indicated by **, and *P* < 0.001 indicated by ***.

## 3. Results

### 3.1. Preparation and Propagation of Neural Tissue Spheres (NTS) Derived from Embryonic Porcine Ventral Mesencephalic (VM) Tissue

Two different tissue preparations for setup of NTS from embryonic porcine VM were tested, namely, microscopic guided manual division of the primary isolated VM tissue block into quarters or sectioning at 350 *μ*m by a McIlwain tissue chopper.

Whereas 85–90% of the manually cut VM tissue blocks rounded up and formed spherical structures within 24 hours in culture, the tissue samples sectioned by tissue chopper had a lower survival rate (40–50%) immediately after establishment and a longer and more protracted recovery period. After 8 days *in vitro* (8 DIV), a large fraction of sectioned VM tissue samples had not rounded up or displayed cavities in the spherical structures. Nonspherical tissue was discarded, and only spherical tissue fragments were included in the study. The sectioning at 350 *μ*m also affected the overall growth rate as documented by prolonged growth periods to reach a passaging diameter of 600–800 *μ*M, as compared to the manually cut NTS. The sectioning did not, however, affect the ability for long-term propagation or the composition of cell types in propagated and differentiated NTS (data not shown). For that reason, we did not—unless specifically mentioned—distinguish between manually or tissue chopper sectioned NTS in the following differentiation experiments.

**Figure 2:**
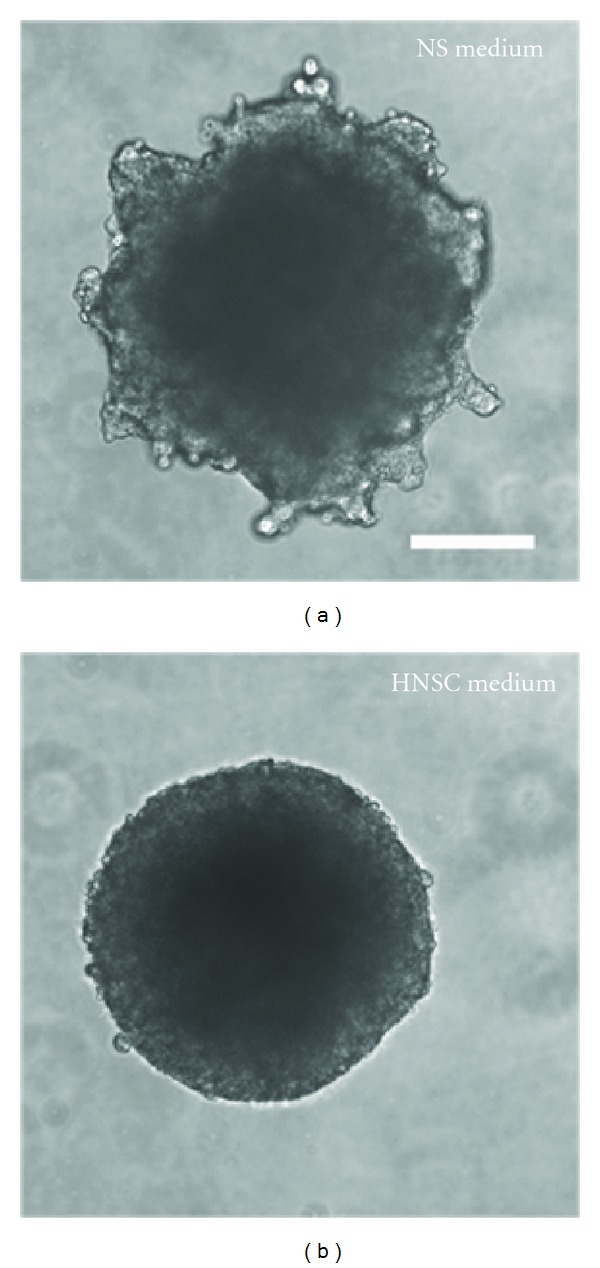
Long-term propagated porcine ventral mesencephalic (VM) neural tissue spheres (NTS). The NTS were propagated in either (a) neurosphere- (NS-) proliferation medium or (b) human neural stem cell- (HNSC-) proliferation medium. Scale bar: 200 *μ*m.

Two different serum-free and mitogen-containing proliferation media, originally developed for culturing of neural stem and progenitor cells, were compared for their ability to stimulate the growth and hence propagation of our porcine NTS. NS-proliferation medium and HNSC-proliferation medium are normally used for murine and human cultures, respectively. The NS-proliferation medium failed to support long-term proliferation of the porcine VM NTS. After 33 days, the proliferative capacity of NTS cultured in NS-proliferation medium was clearly reduced compared to that of NTS cultured in HNSC-proliferation medium. Also the NTS cultured in NS-proliferation medium were unable to form proper spherical structures (Figure 2(a)). As a consequence, use of the NS-proliferation medium was stopped and the cultures excluded from the differentiation study.

In contrast, NTS propagated in HNSC-proliferation medium were viable with display of significant proliferative capacity after propagation for more than 237 days (Figure 2(b)).

**Figure 3:**
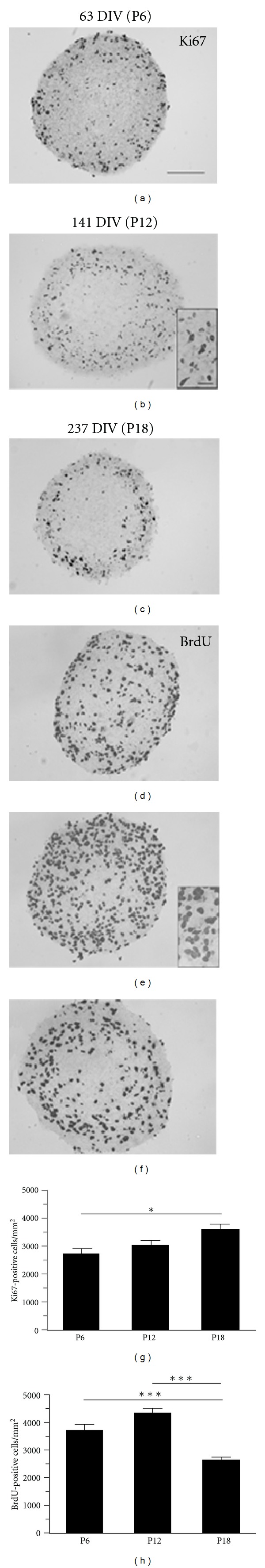
Proliferation in long-term propagated porcine ventral mesencephalic (VM) neural tissue spheres (NTS) visualized by Ki67 and BrdU immunostaining of histological sections. Photomicrographs of NTS showing expression of Ki67 at (a) 63 days *in vitro* (DIV) (passage (P) 6), (b) 141 DIV (P12), (c) 237 DIV (P18) and BrdU labelling at (d) 63 DIV, (e) 141 DIV, and (f) 237 DIV. Scale bars: 100 *μ*m (overview) and 20 *μ*m (insert). Quantification of Ki67 (g) and BrdU (h) expressing cells showed significantly more Ki67-positive cells at P18 as compared to P6, whereas there were significantly more BrdU-positive cells at P6 and P12 as compared to P18. Data are expressed as mean ± SEM (*n* = 15–19; *P < 0.05, ***P < 0.001).

A significant mitotic activity, visualized by Ki67 expression and BrdU incorporation, was observed in both young (P6 = 63 DIV) and long-term propagated cultures (P18 = 237 DIV) (Figure 3). The Ki67 expressing cells predominantly showed a peripheral distribution with the cells forming an outer rim in the NTS (Figures 3(a)–3(c)). The BrdU-positive cells were more numerous and were evenly distributed in the NTS, yet maintaining a slightly higher cell density in the peripheral zones (Figures 3(d)–3(f)). The distribution pattern of BrdU labelled cells suggests some postmitotic/postlabelling migration from the periphery towards the center of the NTS. Quantification of Ki67-positive cells revealed a slight increase in cell densities at higher passages, which was significant at P18 as compared to P6 (Figure 3(g)). Quantification of BrdU-positive cells showed that there were significantly more BrdU-positive cells at P6 and P12 as compared to P18 (Figure 3(h)).

### 3.2. Characterization of Noncultured Foetal Porcine VM Tissue

**Figure 4:**
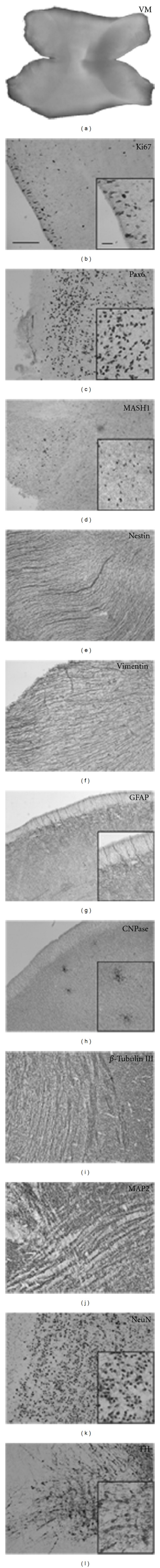
Expression of stem cell markers and markers of glial and neuronal cells in primary (noncultured) porcine ventral mesencephalon (VM) obtained at embryonic day 28–30. Photomicrographs showing an intact VM (a), and immunoexpression of the proliferation marker Ki67 (b), the proneural markers Pax6 (c) and MASH1 (d), the neural stem cell marker nestin (e), the glial markers vimentin (f) and GFAP (g), the oligodendrocyte marker CNPase (h), the neuronal markers *β* Tubulin III (i), MAP2 (j) and NeuN (k), and the dopaminergic marker TH (l). Scale bars: 100 *μ*m (overview) and 20 *μ*m (insert).

To investigate the presence and the composition of neural cell types *in situ* before culturing, freshly isolated (noncultured) embryonic (E28–E30) porcine VM tissue was immunostained for several proliferation and cell-type markers (Figure 4).

In the primary VM tissue, the cell proliferation marker Ki67 was sparsely present with the highest density of cells in the periphery (Figure 4(b)). Pax6, a transcription factor present in proneural cell types, was expressed in round cell nuclei located ventrolaterally to the developing substantia nigra pars compacta and ventral tegmental area (Figure 4(c)). The transcription factor MASH1 was detected only sparsely and found in oval/fusiform cell nuclei distributed in the VM with a tendency of more frequent occurrence lateral to the developing substantia nigra pars compacta and ventral tegmental area (Figure 4(d)).

Cellular expression of the cytoskeletal proteins nestin and vimentin, commonly used for identification of neuroepithelial precursor cells, and GFAP, which is present in both mature astrocytes and some neural stem/progenitor cells, was found widespread in the VM tissue (Figures 4(e)–4(g)).

The oligodendrocyte marker CNPase labelled very few cells in the embryonic porcine VM. The cells present had a “bushy” appearance characteristic of immature oligodendroglia (Figure 4(h)). 

*β* Tubulin III, which is expressed in immature neurons and neuroepithelial cells committed to neuronal fate, was expressed in a large number of cells in the embryonic porcine VM (Figure 4(i)). Cells, positive to the cytoskeletal protein MAP2ab and the nuclear protein NeuN (Figures 4(j)-4(k)), both markers specific to neurons at a certain stage of development or fully mature neurons, were distributed throughout the VM with display of a characteristic neuronal morphology in the MAP2ab staining. TH, which is the rate-limiting enzyme in dopamine synthesis, was expressed in cells displaying a neuronal morphology localized in the developing substantia nigra pars compacta and ventral tegmental area of the VM (Figure 4(l)).

### 3.3. Characterization of Porcine VM NTS after Long-Term Propagation

To address temporal changes of cell-type composition and distribution in the long-term cultured NTS, cells were fixed at days 40, 63, 141, and 237 corresponding to passages 4, 6, 12, and 18, respectively, and immunostained for the same proneural, glial, and neuronal markers as described above for the noncultured VM tissue from embryonic porcine brain.

**Figure 5:**
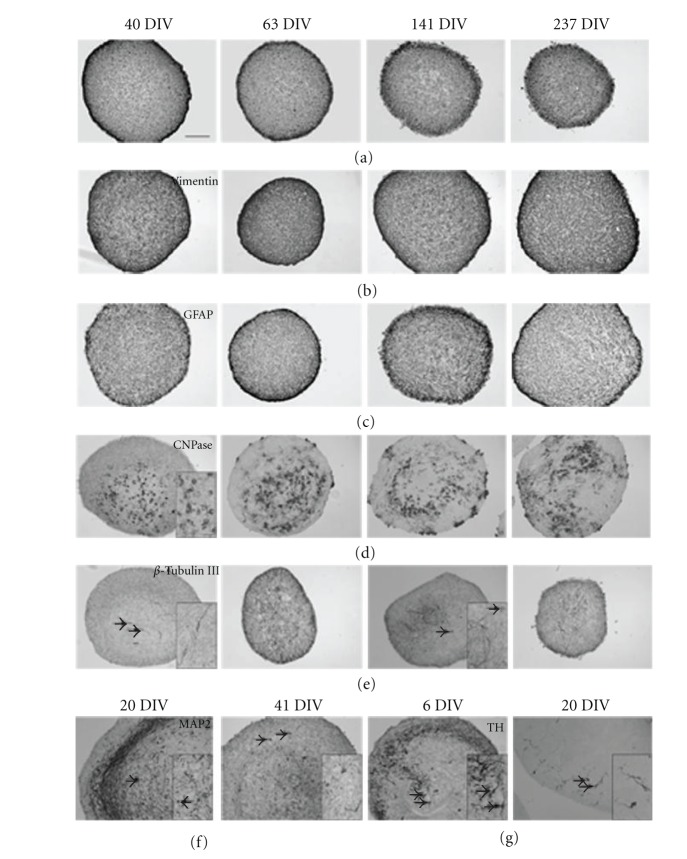
Neural stem cell, glial, and neuronal markers in long-term propagated embryonic porcine ventral mesencephalic, neural tissue-spheres (NTS), stained after 40, 63, 141, and 237 days *in vitro* (DIV). Photomicrographs showing immunoexpression of nestin (a), vimentin (b), GFAP (c), CNPase (d), and *β* Tubulin III (e) in NTS. The neuronal marker MAP2 and the dopaminergic marker TH were not expressed in the long-term propagated NTS, but younger NTS cultures showed MAP2 expression (f) in NTS after 20 and 41 DIV and TH expression (g) after 6 and 21 DIV. Arrows mark neuronal cell soma magnified in inserts. DIV = days *in vitro*. Scale bars = 100 *μ*m (overview) and 20 *μ*m (insert).

Whereas Pax6 and MASH1 expressing cells were observed in the noncultured VM (Figures 4(c)-4(d)), no Pax6- and MASH1-positive cells were identified at any stage in the propagated NTS. In contrast, cells positive for nestin, vimentin, and GFAP were present at high density throughout the NTS at all examined stages of propagation (Figures 5(a)–5(c)).

CNPase-positive cells were identified in clusters in the core of the NTS (Figure 5(d)). The highest density of CNPase-positive cells was observed in the oldest cultures (Figure 5(d); 237 DIV), but the distribution and number of CNPase-positive cells did vary considerably between the individual NTS.

Few *β* Tubulin III-positive cells were seen in the NTS cultures (Figure 5(e)). In cultures grown for 40 days, the *β* Tubulin III-positive cells showed a well-preserved neuronal morphology characterized by a distinct soma with dendritic and axonal ramifications (Figure 5(e); 40 DIV). In older cultures, the morphology was less defined, and primarily *β* Tubulin III-positive cell processes could be identified (Figure 5(e); 141 and 237 DIV).

No NeuN-positive, MAP2ab-positive, or TH-positive cells were observed in the long-term propagated NTS at 63–237 DIV. In contrast to this, both MAP2ab-positive (Figure 5(f)) and TH-positive (Figure 5(g)) cells were present in the newly established NTS (6–41 DIV), while NeuN-positive cells were not identified in newly established NTS either. The data suggest that the fully differentiated neuronal cells in the NTS were eliminated in the NTS with increased propagation time. It could be suspected that this was caused by the use of HNSC-proliferation medium, which “favours” undifferentiated cells, and the passaging technique, which may disrupt the neuronal cell processes and connections.

### 3.4. Differentiation of VM Spheres

Differentiation of the NTS was induced by change to HNSC medium without mitogens and addition of factors known to induce dopaminergic differentiation either *in vitro* in rat (our laboratory; unpublished) or human forebrain cultures [27] or *in vivo* during the normal development of nigral dopaminergic neurons [28, 29]. VM NTS cultures aged between 51 and 232 days were differentiated for 10 or 25 days after plating onto laminin/fibronectin coated plastic plates (Table 1).

Following plating, cells migrated away from the NTS, forming a monolayer of cells arranged as a halo around a central cell mass.

**Figure 6:**
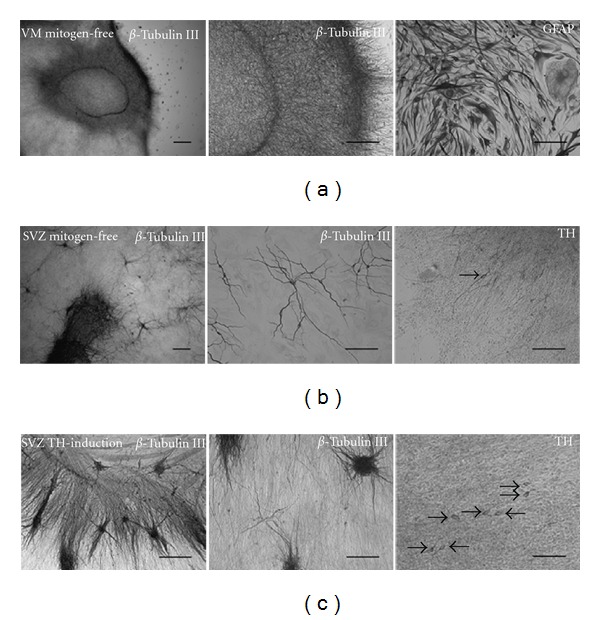
Differentiation of long-term propagated embryonic porcine neural tissue spheres (NTS). Differentiation of VM NTS (51 and 170 days of age) induced by mitogen withdrawal did after 25 days induce a high number of *β* Tubulin III positive cells in the central core of the differentiated NTS (a), but no TH-positive cells were identified. Instead, the differentiated cells primarily expressed the astroglial marker GFAP and displayed a characteristic astroglial morphology. To verify the differentiation potential of the protocols used, 51-day-old NTS cultures derived from the embryonic porcine subventricular zone (SVZ) were included in the differentiation study as a positive control. Differentiation of SVZ NTS for 25 days by mitogen withdrawal (b) or by addition of the TH-inducing factors a-FGF, forskolin, TPA, and db-cAMP (c), known to stimulate TH expression in mammalian forebrain cultures, both resulted in *β* Tubulin III- and TH-positive cells. The *β* Tubulin III-positive cells had a characteristic neuronal morphology and were located in clusters/clones in the zone of migrating cells and in the central core of the differentiated SVZ NTS. The TH-positive cells were more numerous and appeared more morphologically immature by having shorter processes (c) compared to the cultures differentiated under mitogen-free conditions (b). Arrows mark soma of TH-positive cells. Scale bars = 200 *μ*m and 100 *μ*m in microphotographs of *β* Tubulin III expression at low and high magnification, respectively. 100 *μ*m in microphotographs of GFAP and TH expression.

*β* Tubulin type III and TH were used as markers for immature neurons and dopaminergic neurons, respectively. None of the protocols did stimulate dopaminergic differentiation of the VM NTS, as indicated by the absence of TH-positive cells. Instead intense *β* Tubulin type III immunoreactivity was observed in the central cell mass of the plated NTS (Figure 6(a)). Most of the cells in the differentiated VM NTS, however, expressed the astroglial marker GFAP and exhibited characteristic astroglial morphology (Figure 6(a)).

### 3.5. Differentiation of Porcine Subventricular Zone (SVZ) Spheres

Culturing of porcine NTS derived from the SVZ of 28–30-day-old embryos, in either mitogen-free medium or mitogen-free medium supplemented by aFGF, forskolin, TPA, and db-cAMP stimulated *β* Tubulin type III and TH expression, verified a differentiating action of these factors on porcine cultures (Figures 6(b)-6(c)).*β* Tubulin type III-positive cells displaying characteristic neuronal morphology were located both in the halo of migrated cells and in the central cell mass of the differentiated SVZ NTS (Figures 6(b)-6(c)). TH-positive cells were also identified in the differentiated SVZ NTS (Figures 6(b)-6(c)). The TH expressing cells in the mitogen-free cultures were fewer and appeared more mature by having more and longer processes (Figure 6(b)) than the TH-positive cells in the cultures exposed to the TH-inducing factors (Figure 6(c)).

## 4. Discussion

*In vitro* expansion of ventral mesencephalic (VM) precursor cells constitute an interesting alternative to primary fetal VM tissue for intrastriatal transplantation in Parkinson's disease (PD) [8, 30]. Facilitated dopaminergic differentiation of such cells has, however, proven difficult, calling for improved expansion and differentiation protocols.

The domestic pig (*Sus scrofa domesticus*) is an excellent nonprimate large animal model in neuroscience due to the many physiological and biological similarities between human and porcine biology [31, 32]. The pig brain is large enough to provide excellent visualisation, for example, in positron emission tomography and magnetic resonance imaging. Porcine somatic cell nuclear transfer (SCNT) technologies have made the production of transgenic pig models possible [33–35]. Recently, a porcine model for Alzheimer's disease has been developed using “the handmade cloning (HMC) method,” an alternative technique for SCNT, which simplifies the cloning procedure and increases the efficiency [36, 37]. Other porcine models of human brain disorders, like, for instance, the MPTP-induced model of PD, have also been developed [38].

Due to the limited availability of human fetal VM tissue, the pig has been suggested as a nonprimate donor of VM tissue containing dopaminergic neurons and porcine-to-human xenotransplantation of VM tissue has been conducted in a clinical trial [39]. Although immunological rejection processes are induced in human recipient brains by the discordant porcine xenograft, this can be reduced or inhibited by immunosuppression [40]. A very promising application of the new transgenic technology could be the development of genetically engineered pigs for xenotransplantation minimizing graft rejection.

In the present study, porcine VM tissue derived as small tissue pieces from 28–30-day-old embryos was long-term propagated and differentiated as so-called neural tissue-spheres (NTS) [19]. In the NTS system, an *in vivo*-like, organotypic microenvironment is intended to be preserved by avoiding mechanical and enzymatic dissociation thereby maintaining cell integrity, intercellular contact, and cell-cell interaction.

Twenty-eight-day-old embryonic porcine VM shows a certain resemblance to the human VM 6–6.5 weeks postcoitus [41]. TH-positive cells have been identified in the developing human midbrain as early as 3.5 weeks of gestational age, and human fetal VM tissue 6.5–9 weeks of age is considered optimal for transplantation in PD [42, 43]. At 28 days of age, the embryonic porcine VM contains TH-differentiated, dopamine-synthesizing, but otherwise morphologically immature cells with undifferentiated, short processes, capable of surviving and developing *in vitro* and optimal for xenotransplantation in PD patients [41].

Isolated embryonic porcine VM tissue, manually cut into small blocks rounded up and formed NTS within 24 hours, could be maintained and propagated in culture for more than 230 days, using a proliferation medium for human neural stem cells containing the mitogens EGF and bFGF [26]. These results are in good agreement with other studies in our laboratory of long-term culturing of rat and human SVZ and VM as NTS [19, 20, 44]. Here we observed that media used for propagation murine neural stem cells were unable to support long-term propagation of porcine VM NTS.

The NTS method is a modification of the neurosphere techniques originally developed by Reynolds and Weiss [22] and Svendsen et al. [21]. In the neurosphere culture method, passaging by tissue chopping is used successfully for large-scale expansion of nontransformed neural precursor cells isolated from the human cortex. However, our results showed impaired NTS formation when applying a McIlwain tissue chopper instead of manually cutting the porcine VM tissue during passaging. The NTS failed to round up properly. Our observations thus indicate a higher sensitivity to mechanical stress of porcine brain tissue compared to human tissue and/or midbrain tissue versus forebrain tissue.

### 4.1. Cellular Content of Propagated NTS

Using cell proliferation and cell-type markers, the proliferative activity and cell-type composition of NTS were evaluated at defined propagation stages of porcine VM NTS.

In contrast to a sparse occurrence of proliferating cells in the primary VM tissue identified by Ki67, a significant proliferative activity was visualized by Ki67 immunostaining and BrdU labelling in both young (P6 = 63 DIV) and old VM NTS cultures (P18 = 237 DIV). The occurrence of the dividing Ki67-positive cells were primarily confined to the peripheral part of the NTS, while BrdU-positive nuclei were more evenly distributed in the NTS, even that the highest cell density was found peripherally.

Ki67-expression reflects mitotic activity at the time of fixation, while BrdU incorporation covers a wider time span corresponding to the 24-hour exposure period. Several BrdU-positive cells are therefore expected to be postmitotic at the time of fixation. In the superficial parts of the NTS, the nutritional condition and oxygen level might be supportive for cell proliferation, while the BrdU-positive cells located in a more central part of the NTS are likely to be postmitotic cells that have migrated into the deeper parts of the NTS but may also represent cells undergoing DNA repair [45].

Previous data from our laboratory have shown a change from a peripheral distribution of Ki67- and BrdU-positive nuclei in young (P2 = 35 DIV) NTS, derived from foetal rat VM to a more evenly distribution in older NTS [20]. The rat VM NTS were propagated in media containing leukemia inhibitory factor (LIF) and bFGF, instead of EGF and bFGF, and this might explain the discrepancy between the spatial distribution of proliferative cells in long-term propagated porcine and rat VM NTS. Differences in culture treatment might also have influenced the outcome.

The proneural transcription factors Pax6 and MASH1 are essential in both fetal and adult/postnatal neurogenesis in the mammalian brain. Pax6 and MASH1 are expressed by neural stem/progenitor cells in the two adult brain neurogenic zones, that is, the SVZ of the lateral ventricle and the subgranular zone of the hippocampal dentate gyrus [46–48]. The factors seem to function in the early steps to balance proliferation and differentiation of neural stem cells.

In this study, the proneural markers Pax6 and MASH1 were identified in the porcine primary VM tissue corresponding to start of culturing, but were not found in the cultured VM NTS at any stage of propagation. Using RT-PCR analyses, Kukekov et al. [49] have previously shown the presence of Pax6 RNA transcripts in neurosphere cultures derived from the periventricular subependymal zone and the hippocampus of the adult human brain (age 24–57 years).

Expression of structural proteins like nestin (neuroepithelial stem cell protein) and vimentin, which both are intermediate filament proteins, is used for positive identification of neuroepithelial stem cells [50, 51]. Recently, another cytoskeletal protein, GFAP (glial fibrillary acid protein), formerly believed to be expressed exclusively in differentiated glial cells, has been identified in neural stem/progenitor cells in the adult mammalian SVZ and hippocampus [52, 53]. Nestin, vimentin, and GFAP expression have been found to be expressed in neural progenitor cells isolated from the postmortem human cortex [54].

In our NTS system, nestin, vimentin, and GFAP proteins were widely expressed in both the primary VM tissue as well as in the NTS cultures during the entire long-term propagation period. Nestin, vimentin, and GFAP-positive cells were evenly distributed in the NTS and no decrease in the intensity of the immunostaining was seen with increasing age of the cultures. Mature astrocytes also express GFAP and the pool of GFAP-positive cells therefore must include fully differentiated glia, at least in the younger NTS.

During the long-term propagation period (237 DIV), the porcine VM NTS displayed an increase in number of CNPase-positive cells with structural resemblance to oligodendrocytes appearing in clusters primarily in the central core of the cultures. The cells confirm the presence of a core microenvironment sufficient in nutrition and oxygen for maintaining cell metabolism.

Only few CNPase expressing cells were identified in the primary VM tissue before culturing. The temporal increase in the number of oligodendrocytes in the cultured NTS probably reflects an intrinsic histogenetical programming of the VM tissue, which persists in the *in vitro* culture conditions. It is well known that oligodendroglia appear later than neurons in the histogenesis of the nervous system and continues to develop postnatally [55].

Besides neural precursor cells and differentiated glial cells, the acutely isolated E27–30-day-old porcine VM tissue contained mature and immature neurons verified by the expression of the MAP2 (mature), TH (dopaminergic), and *β* Tubulin III (immature) neuronal markers. Whereas immature neuronal cells expressing *β* Tubulin III could be identified in both young and old NTS, the mature neurons died off within the first passages of the NTS (MAP2 = 42 DIV; TH = 21 DIV) in the presence of a serum-free, bFGF, and EGF containing proliferation medium, leaving only the stem/progenitor cells to proliferate. The sustained expression of *β* Tubulin III might be the result of a spontaneous neuronal differentiation of precursor cells within the propagated NTS previously demonstrated in the classical neurosphere assay [56].

### 4.2. Induced Differentiation of VM NTS

Studies have shown that the neuronal outcome of differentiation of intact neurospheres is higher compared to the outcome from dissociated cultures and that differentiation at high cell densities yields more neurons compared to differentiation at low densities, demonstrating the importance of intercellular contact during neuronal development [18, 57].

In the present study, porcine VM NTS, aged 51–232 DIV, were plated onto laminin-coated plastic plates and left to differentiate for 10 or 25 days in either mitogen-free medium or mitogen-free medium supplemented by aFGF, forskolin, TPA, and db-cAMP or FGF-8b and SHH. 

Whereas removal of mitogens from the culture medium induces a noncell-type-specific differentiation, addition of the factors aFGF, forskolin, TPA, and db-cAMP induces tyrosine hydroxylase (TH) expression in rat forebrain cultures and cultures of the NT2/hNT cell line, derived from human teratocarcinoma cells [29]. The factors seem to stimulate TH expression by activating the protein kinase A (PKA) and protein kinase C (PKC) signalling pathways [29].

Shh and FGF-8b are essential inducers of dopaminergic differentiation at specific induction sites in the midbrain and forebrain in rat embryos [28]. Results from our laboratory have also demonstrated a mitogen effect of FGF-8b on midbrain dopaminergic precursor cells from rat embryos [9, 58].

None of the protocols tested in this study stimulated dopaminergic differentiation/TH expression in the porcine VM NTS, but did induce *β* Tubulin type III expression. Most of the cells in the differentiated VM NTS expressed the astroglial marker GFAP and exhibited characteristic astroglial morphology.

When porcine subventricular zone (SVZ) NTS derived from E28–30-day-old embryos were grown in either mitogen-free medium or mitogen-free medium containing aFGF, forskolin, TPA, and db-cAMP, the expression of *β* Tubulin type III and TH was enhanced verifying the relevance of applying these protocols to porcine cultures. Speculatively, it may be suggested that aFGF, forskolin, TPA, and db-cAMP are forebrain-specific inducers of dopaminergic differentiation, which, however, are unable to induce dopaminergic differentiation of midbrain-derived precursor cells.

FGF-8b and SHH are key factors in the development of midbrain dopaminergic cells *in vivo*, but might be inadequate for induction of the dopaminergic phenotype *in vitro*. For instance, cotreatment with FGF-8b and SHH only has a minor TH-inductive effect (less than 5%) on mouse embryonic striatal precursor cells and the human stem cell line NT2/hNT *in vitro* [59]. Of course, the discrepancies between the *in vivo* and *in vitro* data could reflect differences between the origin and the developmental stage of the cell types studied, but an alternative and plausible interpretation might also be that one or more additional mediators are essential for induction and specification of the midbrain dopaminergic neuron *in vivo*. One such molecule could be transforming growth factor-*β* (TGF-*β*), which has been shown to be essential for the induction and maintenance of midbrain dopaminergic neurons both *in vitro* and *in vivo* in chicken and rat models [60].

Even without a direct dopaminergic potential, the NTS might still be relevant for transplantation in PD. A recent study by Moses et al. [61] has shown that long-term propagated neurospheres derived from the mouse foetal striatum and ventral mesencephalon are capable of rescuing dopaminergic neurons from induced apoptosis and promoting survival *in vitro* of dopaminergic neurons and *in vivo* of the nigrostriatal system probably by soluble contact independent factors. 

In summary, our data show for the first time that ventral mesencephalic (VM) precursor cells, isolated from porcine embryos 28–30 days of age, can be long-term propagated as neural tissue-spheres (NTS). After more than 230 days, the NTS still form neurons and glia, thus allowing experimental studies of neural precursor cells, their niche and differentiation capacity.

## Supplementary Material

List of primary antibodies used for immunohistochemistry

## Figures and Tables

**Table 1:** Differentiation of long-term propagated neural tissue-spheres (NTS) derived from the embryonic porcine ventral mesencephalon (VM).

Age (DIV)	Passage (P)	Protocol(s)	Duration (days)
51		(1) Mitogen-free	
	P2	(2) a-FGF, forskolin, TPA, db-cAMP	25
170		(1) Mitogen-free	
	P14	(2) a-FGF, forskolin, TPA, db-cAMP	10
232		(1) Mitogen-free	
	P18	(2) FGF8 + sonic hedgehog	10
