# Supplementary material for: Characterization of Porcine Ventral Mesencephalic Precursor Cells following Long-Term Propagation in 3D Culture
Source: Stem Cells Int. 2012 Nov 20;2012:761843. doi: 10.1155/2012/761843 (PMC3508616; doi:10.1155/2012/761843)
Supplement: Supplementary file 1 — List of primary antibodies used for immunohistochemistry [file 761843.f1.doc]

Supplementary Table 1 – Primary antibodies used

| **Marker/antigen** | **Species(subtype)** | **Clone** | **Manufacturer** | **Product code** | **Dilution** |
| --- | --- | --- | --- | --- | --- |
| **Proliferation** |  |  |  |  |  |
| BrdU | monoclonal mouse (IgG1) | Bu20a | DAKO | M 0744 | 1:200 |
| Ki67 | monoclonal mouse (IgG1) | B56 | BD Biosciences | 550609 | 1:200 |
| **Neural stem cell** |  |  |  |  |  |
| Nestin | polyclonal rabbit (IgG – affinity purified) |  | Abcam | Ab5968 | 1:500 |
| **Proneuronal** |  |  |  |  |  |
| Pax 6 | polyclonal rabbit - serum |  | Chemicon | AB5409 | 1:2000 |
| Mash 1 | monoclonal mouse (IgG1) | 24B72D11.1 | BD Biosciences | 556604 | 1:50 |
| **Neuronal** |  |  |  |  |  |
| β-tubulin III | monoclonal mouse (IgG2b) | SDL.3D10 (ascites fluid) | Sigma | T8660 | 1:2000 |
| MAP2 (a+b+c) | monoclonal mouse (IgG1)  monoclonal mouse (IgG1) | AP18  HM-2 | Neomarkers  Sigma | MS-250  M4403 | 1:500  1:2000 |
| NeuN | monoclonal mouse (IgG1) |  | Chemicon | MAB377 | 1:500 |
| **Dopaminergic** |  |  |  |  |  |
| TH | polyclonal rabbit - purified |  | Pel-Freez | P40101-0 | 1:1000 |
| **Macroglial** |  |  |  |  |  |
| Vimentin | monoclonal mouse (IgG1) | V9 | DAKO | M 0725 | 1:2000 |
| GFAP | polyclonal rabbit - purified |  | DAKO | Z 0334 | 1:5000 |
| CNP’ase | monoclonal mouse (IgG1) | 11-5B | Chemicon | MAB326R | 1:400 |
